# Unboiled and Boiled Milk

**Published:** 1886-10

**Authors:** 


### *BOILED AND UNBOILED MILK.*

The inability or the disinclination of the modern woman to supply to her young its natural pabulum, is the most fruitful cause of the high percentage of mortality in infants under two years of age. The ingenuity of man has united his knowledge of physiology and chemistry, to supply a substitute for the natural secretion, and we have in the market a large variety of artificial foods for infants. While some of these are tolerably successful, few, if any, are equal to the milk of the cow, which, with slight modification and with proper care in its preservation, may be made to quite closely conform to human milk. In spite, however, of the most scrupulous care, raw milk is most frequently illy borne.

"Recent experiments by Dr. Reichmann (*Deutsch Med. Zeitung*), seem to show that by boiling the milk these difficulties may be obviated. The following are the results of these experiments:

"1. Boiled milk leaves the healthy stomach more rapidly than an equal quantity of unboiled milk.

"2. The digestion of boiled milk is more rapidly accomplished than that of unboiled milk.

"3. The coagulation of unboiled milk in the stomach is complete in five minutes.

"4. The coagulation is not caused by the acid of the gastric juice, but by the influence of a special milk curdling ferment.

"5. The acidity of the gastric juice is, at first, due almost solely to the lactic acid, and, later in the process of digestion, to the presence of hydrochloric acid.

"6. Hydrochloric acid first appears in perceptible amount, forty-five minutes after the ingestion of half of a pint of milk.

"7. For the first hour and a quarter, after the ingestion of milk, the acidity gradually increases, then decreases until the milk has entirely left the stomach.

"8. The curds of casein, in the digestion of boiled milk, are much softer than in the case of uncooked milk.—*Med. Age.*"

---

In the transactions of the Chicago Medical Society, for May, Dr. W. T. Belfield is credited with presenting specimens of the *anchylostomum duodenale*, a parasite of the nematoid variety, taken from the intestine of a cat, which died from anæmia. Attention was first called to this worm in 1838, by Duteni, of Milan, but its true pathalogical significance remained to be explained by Griesinger in 1851, who identified it as the primary agent in the causation of persistent anæmia and chlorosis, so common in the East Indies, and the warmer counties of both the eastern and western continents.

Numerous instances are recorded by German writers of their presence in the small intestine of both human subjects and those of various animals, dissected in laboratory work. In appearance they resemble somewhat the small thread worm. It is about 8 to 10 lines in length, and has a cone-shaped head, from which projects four tenacula. By means of these they fasten themselves to the mucous lining of the intestine, and extract the blood of their unfortunate victim.

In addition to the chlorotic state, which characterizes their presence, tympanties and pain is generally found over the small intestine, and in the later stages, watery stools, often containing blood, are present. All efforts to obtain specimens of this parasite from the stools have, as far as can be learned, been unsuccessful.

In Brazil, where the disease is quite frequent, the pulp of fresh figs is said to rapidly effect a cure. An alcoholic extract prepared from the juice of the *ficus doliaria*, a variety of fig found in Brazil, and known as doliarina is considered the best anthelmintic to be employed, and is administered in conjunction with iron and other remedies, to combat the marked anæmia, always so characteristic of diseases in which the primary cause is found to be some vitiated condition of the blood.

---

HENRY HERRMANN, long known as a manufacturer of Surgical and Orthopædical Instruments, has wisely determined on the removal of his factory and store to a location more convenient for physicians. On or about October 15th he will remove to No. 9 West Huron street. The store is being thoroughly renovated for his special wants, and when finished, it will be the most complete establishment of the kind in Western New York. With the latest improved machinery he will be enabled to fill all orders at lowest rates. Mr. Herrmann's practical ability in the mechanical treatment of all deformities of the human frame and hernia, also of the manufacture of surgical instruments and cutlery, is generally recognized.

